# Assessing the Quality and Accuracy of ChatGPT-3.5 Responses to Patient Questions About Hip Arthroscopy

**DOI:** 10.3390/medicina61122080

**Published:** 2025-11-22

**Authors:** Maximilian Heinz, Hassan Tarek Hakam, Mikhail Salzmann, Robert Prill, Nikolai Ramadanov

**Affiliations:** 1 Center of Orthopeedics and Traumatology, University Hospital Brandenburg/Havel, Brandenburg Medical School, Hochstraße 29, 14770 Brandenburg an der Havel, Germanynikolai.ramadanov@gmail.com (N.R.); 2 Faculty of Health Science, Brandenburg Medical School, 14770 Brandenburg an der Havel, Germany

**Keywords:** artificial intelligence, large language models, patient education as topic, femoroacetabular impingement, hip, arthroscopy, orthopedics

## Abstract

*Background and Objectives*: Artificial intelligence-driven large language models such as ChatGPT increasingly influence patient education in surgical fields. This study evaluates the quality and accuracy of ChatGPT-3.5-generated responses to patient questions regarding femoroacetabular impingement syndrome (FAIS) and hip arthroscopy (HAS). *Materials and Methods*: In this descriptive observational study, ChatGPT-3.5 generated and answered 20 representative patient questions about FAIS and HAS (n = 20 question–answer pairs). No patient-derived questions or data were used. Each response was independently evaluated by two fellowship-trained orthopedic surgeons across four domains: relevance, accuracy, clarity, and completeness, using a five-point Likert scale. Inter-rater reliability was calculated using the intraclass correlation coefficient (ICC), and descriptive inter-rater agreement percentages were reported. Additional qualitative impressions from the reviewers were recorded to contextualize areas in which responses were rated slightly lower, particularly regarding explanatory depth and postoperative variability. *Results*: Mean ratings across all domains ranged from 4.85 ± 0.24 (95% CI: 4.74–4.96) to 5.00 ± 0.00. Relevance achieved a perfect mean score of 5.00, while accuracy and clarity each obtained 4.98 ± 0.11 (95% CI: 4.91–5.00). Completeness demonstrated the lowest scores (4.85 ± 0.24). Due to pronounced ceiling effects, ICC values were non-informative; however, descriptive agreement between raters was high, with 100% concordance for relevance and 90% agreement for accuracy and clarity. No factually incorrect or unsafe information was identified. Overall, responses were concise, structured, and clinically appropriate, though occasionally lacking in granularity concerning individual recovery trajectories and procedure-specific nuances. *Conclusions*: ChatGPT-3.5 demonstrates significant potential as a supplementary patient education tool in hip preservation surgery. While its responses were consistently accurate and easy to understand, their occasional lack of detail, particularly concerning postoperative expectations and variability in outcomes, indicates that the findings apply primarily to synthetic, standardized questions in a controlled setting. Further validation is required before generalizing these results to real-world patient interactions. Future studies should incorporate authentic patient questions, diverse evaluator groups, and longitudinal assessment across different LLM versions to better define clinical applicability and safety.

## 1. Introduction

FAIS is a major cause of hip pain in young and middle-aged adults, with an incidence of 54.4 per 100,000 person-years [1]. It results from abnormal contact between the acetabulum and femur, which can cause labral tears, cartilage damage, and early osteoarthritis. HAS has become the preferred treatment, achieving favourable outcomes and fewer complications than open approaches [2,3,4,5,6]. Its minimally invasive nature enables correction of labral and bony pathology while preserving joint function and shortening recovery [7,8]. In recent years, the overall number of hip arthroscopic procedures performed worldwide has continued to rise, reflecting both advances in surgical technique and increasing awareness of FAIS among clinicians and patients alike.

As HAS usage grows worldwide, patients increasingly seek reliable information about their condition before consultation. Nearly 40% of orthopaedic patients now search online for health information, reflecting a broader trend toward patient empowerment and shared decision-making [9]. However, the accuracy and readability of online content remain inconsistent, posing a risk of misinformation. This issue is particularly relevant for younger, active individuals with FAIS, who often rely heavily on digital sources when evaluating treatment options, including whether surgery is necessary and what outcomes they may realistically expect.

The emergence of large language models (LLMs) such as ChatGPT marks a major shift in how patients access medical knowledge [10]. Offering natural, conversational responses, ChatGPT has achieved accuracy rates between 52.1% and 92%, depending on specialty and question complexity [11,12]. In orthopaedics, ChatGPT-4.0 has produced generally satisfactory answers regarding joint arthroplasty, with accuracy up to 76.7% [13,14,15,16,17]. Despite this promising performance, its output varies substantially depending on the structure of the question, the level of clinical nuance required, and the availability of condition-specific information in its training data.

Yet, its performance in FAIS and HAS contexts remains insufficiently characterized. Özbek et al. found that 88% of ChatGPT-4.0 responses to common HAS questions were rated “excellent” or “satisfactory requiring minimal clarification” by expert reviewers [18]. Nevertheless, concerns persist about incomplete information, lack of clinical nuance, and poor readability beyond recommended literacy levels [19]. These limitations may reduce the practical usefulness of LLM-generated content for patients making decisions about hip arthroscopy, where understanding anatomy, procedural details, and postoperative expectations is crucial for informed consent.

AI-driven chatbots offer both promises and challenges for orthopaedic education. While they may enhance patient understanding and accessibility [20], risks of misinformation, bias, and unequal performance across populations remain [9,12,21,22]. As digital health literacy becomes increasingly crucial, evaluating the quality and reliability of AI-generated information for patient education in HAS is essential [23,24,25]. Moreover, the rapid evolution of LLM technology underscores the need for continuous reassessment, as model performance may change substantially within short development cycles.

Although more advanced LLMs such as GPT-4 and GPT-4o have recently demonstrated improved reasoning and clinical accuracy, ChatGPT-3.5 remains highly relevant for patient-facing contexts because it is freely accessible and thus the version most commonly used by the general public. Evaluating ChatGPT-3.5 therefore provides a realistic appraisal of the information patients are most likely to encounter, rather than reflecting the performance of subscription-based premium models. Its assessment also establishes a practical baseline against which future model advances can be compared. By examining the clarity, completeness, and clinical appropriateness of ChatGPT-3.5 responses to FAIS- and HAS-related questions, this study aims to determine whether the information provided by freely accessible LLMs is suitable for patient education. This study investigates ChatGPT-3.5’s accuracy and clinical appropriateness in responding to patient questions about FAIS and HAS, as assessed by fellowship-trained orthopaedic surgeons.

## 2. Materials and Methods

### 2.1. Study Design and Data Generation

This descriptive observational study evaluated the educational content generated by ChatGPT-3.5 (OpenAI) for patients with FAIS and HAS. Data generation was conducted via the ChatGPT web interface (https://chat.openai.com, accessed on 10 March 2025) between 10 and 12 March, 2025, using the default GPT-3.5 model settings (temperature = 1.0, maximum tokens = default, no custom system prompt). The model was accessed in English (United States) under default regional settings. In the initial query, the model was instructed to “Create a list of the 20 most frequently asked questions by patients about femoroacetabular impingement syndrome and hip arthroscopy.” ChatGPT-3.5 produced 20 distinct questions, which were recorded verbatim and presented in Table 1. Subsequently, each of these questions was entered in a separate chat session with the prompt, “Please answer this question in a clear, patient-friendly manner.”

### 2.2. Response Collection

All responses were automatically generated by ChatGPT-3.5 and documented exactly as produced, without any manual modification, editing, or correction, to preserve the authenticity of the model’s outputs. The complete responses are provided in the Supplementary Materials (Supplementary Table S1).

### 2.3. Evaluation Protocol

Two independent fellowship-trained orthopaedic surgeons (N.R. and H.T.H.) with expertise in hip preservation surgery independently evaluated the 20 ChatGPT-3.5-generated responses. Each response was evaluated across four predefined domains using a slightly modified rating scale based on Magruder et al. [26], assessing relevance, accuracy, clarity, and completeness. Prior to the formal rating, a brief calibration phase was conducted in which both raters jointly reviewed exemplar responses to harmonize their interpretation of scores, particularly for differentiating between 4 (largely appropriate with minor omissions) and 5 (excellent, complete coverage). Relevance was defined as the extent to which the response addressed the corresponding question. Accuracy referred to the factual correctness of the provided information. Clarity was evaluated based on comprehensibility and linguistic precision, while completeness referred to the extent to which all essential aspects of the topic were covered Each domain was rated on a five-point Likert scale (1 = poor performance, 5 = excellent performance). To enhance interpretability, intermediate scores were explicitly defined. A rating of 4 was assigned when the response was largely appropriate with only minor omissions or limited nuance. A score of 3 indicated adequate but incomplete coverage, with notable missing details or oversimplification. A score of 2 reflected substantial gaps, ambiguity, or insufficient depth that could compromise patient understanding. A score of 1 indicated poor performance with major inaccuracies or omissions. Both evaluators were blinded to each other’s ratings and instructed to flag any content considered factually incorrect, clinically unsafe, or misleading. Both reviewers completed their assessments independently and were blinded to each other’s ratings. No adjustments or consensus ratings were made after submission of the individual scores.

### 2.4. Statistical Analysis

For each evaluation domain, the mean and standard deviation (SD) were calculated across all 20 items. Inter-rater reliability was assessed using a two-way random-effects intraclass correlation coefficient with absolute agreement and single-measure definition [ICC(2,1)], following the framework proposed by McGraw and Wong [27]. This model was selected because both raters were considered random representatives of a broader population of potential evaluators, and each item was rated by both reviewers, making a two-way random-effects approach appropriate. Absolute agreement was used to quantify the extent to which both raters assigned identical scores rather than merely consistent rank ordering. Because ICC values may become unstable in the presence of minimal between-item variance (a known issue in datasets with pronounced ceiling effects), descriptive agreement—defined as the exact percentage of identical ratings between the two raters (N.R. and H.T.H.)—was also reported to provide a more interpretable measure of consistency. Although the Likert ratings represent ordinal data, they were treated as continuous for ICC estimation, consistent with standard practice in psychometric agreement analyses. To account for the ordinal nature of the scale, medians and interquartile ranges for each domain were additionally calculated as a sensitivity analysis. All statistical analyses were performed using Python software (version 3.12; Python Software Foundation, Wilmington, DE, USA), including the libraries pandas for data handling, numpy for numerical operations, and pingouin for ICC computation.

## 3. Results

### 3.1. Descriptive Statistics

Mean ratings for each evaluation domain are summarized in Table 2. Overall, the scores were uniformly high across all four domains, based on 20 question–answer pairs independently evaluated by two expert raters. Mean values ranged from 4.85 ± 0.24 (95% CI: 4.74–4.96) to 5.00 ± 0.00. Relevance achieved a perfect mean score of 5.00 across all items, while accuracy and clarity each demonstrated nearly identical results (4.98 ± 0.11, 95% CI: 4.91–5.00).

Completeness showed the greatest variability (4.85 ± 0.24, 95% CI: 4.74–4.96). The complete summary of quantitative ratings and agreement metrics is presented in Table 3.

### 3.2. Inter-Rater Reliability and Agreement

Because the distribution of ratings exhibited a pronounced ceiling effect, intraclass correlation coefficient (ICC) values were not informative, yielding estimates close to zero or non-estimable results - a known limitation when evaluating highly skewed Likert-scale data. Despite this statistical constraint, descriptive agreement between the two raters (N.R. and H.T.H.) was high across all domains: complete agreement for relevance (100%) and strong agreement for accuracy (90%), clarity (90%), and completeness (70%).

The descriptive results, as presented in Table 2, are supported by the item-level ratings shown in Table 3. Most scores were 5 across all domains, with only a few 4s observed, particularly for completeness. Specifically, for questions 4–20, 12 of 17 completeness pairs were identical between raters, corresponding to approximately 70.6% exact agreement, which aligns with the rounded 70% reported in Table 2. Notably, the few disagreements in completeness scores predominantly occurred for questions addressing postoperative expectations and long-term outcomes, reflecting the clinical complexity of these topics.

Overall, these findings demonstrate consistently high performance across all domains and substantial consensus between raters. Despite the limitations of ICC estimation in this dataset, the descriptive agreement percentages, supported by item-level patterns, provide a reliable measure of inter-rater consistency and highlight areas where responses were slightly less comprehensive.

### 3.3. Content Quality Assessment

Evaluation of the scores provided by the two fellowship-trained orthopedic surgeons demonstrated uniformly high ratings across all domains, indicating that ChatGPT-3.5’s responses were of consistently high quality with only minor areas for improvement. The lowest score assigned in any category was four out of five, underscoring the overall strength of the model’s performance. Prior to the evaluation, raters were instructed to flag any content they considered factually incorrect, misleading, or clinically unsafe. However, no such instances were identified during the assessment. In general, ChatGPT-3.5’s answers to the 20 patient-oriented questions were comprehensive and patient-friendly, frequently concluding with recommendations to consult a treating orthopedic surgeon or physical therapist for individualized advice. The responses were presented in a logically structured and well-organized format, often distinguishing between diagnostic, therapeutic, and prognostic aspects while emphasizing that personalized recommendations may vary depending on clinical context.

Across all domains, mean scores ranged from 4.85 ± 0.24 to 5.00 ± 0.00, reflecting minimal variability. The highest mean values were observed for accuracy and clarity (4.98 ± 0.11 each), followed closely by relevance, which achieved a perfect mean of 5.00 across all items. Completeness demonstrated relatively lower scores (mean 4.85 ± 0.24), suggesting that while ChatGPT-3.5 provided clear and factually correct explanations, occasional omissions of detailed or quantitative information limited full comprehensiveness.

### 3.4. Qualitative Findings

Qualitatively, both raters noted that the model maintained an appropriate tone for patient education, avoided overly technical language, and accurately summarized key elements of FAIS and HAS. The responses were well organized and contextually appropriate. Occasional brevity in describing aspects such as surgical indications, the underlying pathophysiology, diagnostic evaluation, procedural details of hip arthroscopy, or expected timelines for recovery and return to activity accounted for the slightly lower completeness scores. Despite these minor omissions, the answers remained accurate, clear, and safe, with no misleading or factually incorrect information identified in any of the generated responses.

## 4. Discussion

### 4.1. Primary Findings

This study demonstrated that ChatGPT-3.5 produced highly relevant, accurate, and comprehensible responses to common patient questions regarding FAIS and HAS. Both fellowship-trained orthopaedic surgeons rated the AI-generated content consistently high across all evaluation domains, with mean scores exceeding 4.8 out of 5. The uniformly elevated ratings indicate that ChatGPT-3.5 provided patient-centered, evidence-informed explanations with only minimal omissions or ambiguities. Importantly, the lowest score assigned in any category was 4, underscoring the model’s ability to generate high-quality educational content without introducing factual inaccuracies or misleading information. It should be noted, however, that the evaluation was based on only 20 synthetic questions and did not include validation of readability or comprehension by patients or lay readers, which may limit the generalizability of the findings. Beyond these considerations, the consistently strong performance across numerous domains suggests that ChatGPT-3.5 may already be capable of supporting foundational stages of patient education, particularly for individuals seeking introductory explanations prior to formal clinical consultation. This aligns with broader evidence indicating that early informational exposure can shape patient expectations, reduce anxiety, and facilitate more productive shared decision-making.

### 4.2. Consistency and Inter-Rater Agreement

Both reviewers consistently rated ChatGPT-3.5’s responses as highly relevant, accurate, and clear, with minimal variability between items. The observed ceiling effect (mean > 4.8) limited the interpretability of intraclass correlation coefficient (ICC) values but simultaneously reflected near-complete agreement and overall consistency in qualitative judgment. Although ICC estimates were statistically non-informative, this limitation arises from uniformly high performance rather than a lack of evaluator consensus. Such findings suggest that, within the context of patient education, ChatGPT-3.5’s content generation can achieve a level of reliability comparable to expert-reviewed materials when evaluated under structured conditions. Moreover, the tight clustering of ratings highlights that both reviewers interpreted the evaluation criteria similarly, reinforcing the internal coherence of the scoring framework used in this study. This degree of concordance is noteworthy because clinical experts often diverge in their emphasis on completeness versus conciseness when judging educational materials, yet here their assessments aligned almost perfectly. The limited score variation therefore reflects the stability and predictability of the model’s outputs rather than any methodological constraint.

### 4.3. Comparison with Prior Studies

The high ratings observed across all four domains are consistent with findings from prior research on ChatGPT’s performance in orthopaedic contexts. Özbek et al. reported that ChatGPT-4.0 achieved predominantly “excellent” ratings when evaluated on similar HAS-related questions, with only minimal clarifications required. Similarly, Sparks et al. found that ChatGPT-3.5 provided generally accurate information about orthopaedic procedures, though at times lacking in depth or individualized context. It is important to note that these prior evaluations often used questions derived from real patient inquiries, whereas the present study assessed model-generated questions designed to represent typical patient concerns. The current study extends these observations to ChatGPT-3.5, demonstrating consistent refinement in linguistic clarity and factual precision across successive evaluations while highlighting the utility of synthetic question sets for structured, reproducible assessment. In contrast to earlier reports, our findings show that ChatGPT-3.5 has become increasingly stable in its explanation patterns, providing not only accurate but also stylistically coherent responses. This may reflect progressive optimization of the underlying training data as well as incremental improvements introduced by the model developers. Additionally, the strong alignment with prior studies strengthens the emerging consensus that LLMs can reliably convey anatomical and procedural concepts in musculoskeletal medicine.

### 4.4. Limitations and Clinical Implications

Despite these favorable results, certain limitations inherent to AI-generated content remain evident. Completeness received slightly lower mean ratings compared to other domains, primarily due to occasional brevity in postoperative details or prognostic variability. This suggests that while ChatGPT-3.5 effectively summarizes core concepts, it may not consistently capture the full clinical spectrum or provide nuanced discussions of patient-specific factors. Moreover, several methodological limitations should be acknowledged. First, the evaluation was performed using a single LLM version (ChatGPT-3.5) at one time point, which may not reflect performance changes over successive updates or across newer models. Second, no patient cohort was included to assess actual comprehension, retention, or behavior change, limiting the ability to infer real-world impact. Third, the study relied on only two fellowship-trained orthopaedic raters without input from a broader expert panel or non-orthopaedic perspectives, which may affect the generalizability of the assessments. Additionally, the list of questions was generated by ChatGPT-3.5 rather than derived from real patient inquiries or survey data. Consequently, both the informational prompts and their corresponding responses originated from the same algorithmic source, introducing potential circularity. While this approach ensured internal consistency, it may limit external validity, since naturally occurring patient concerns could differ in complexity, phrasing, or emphasis. Similar patterns have been described in previous studies evaluating AI chatbots in surgical education, where high factual accuracy coexisted with limited contextual or individualized depth. Therefore, while ChatGPT-3.5 can serve as an efficient introductory resource, its outputs should supplement rather than replace professional consultation and individualized patient education. Future research should explore multiple LLM versions over time, include patient-centered evaluations of comprehension and decision-making, and incorporate broader expert panels to better assess content quality and applicability. Furthermore, integrating real patient feedback may reveal discrepancies between expert-based judgments and lay understanding, particularly in relation to medical terminology, risk communication, and expectations regarding recovery timelines. Another important consideration is the potential for subtle inaccuracies or oversimplifications to influence patient beliefs—effects that may not be immediately detectable through expert review alone. As AI systems become more embedded in patient education, establishing mechanisms for clinician oversight, content verification, and continuous quality assurance will be essential to maintain safety and trust.

### 4.5. Interpretation of Statistical Findings

The low inter-rater reliability metrics observed in this study should be interpreted cautiously. The near-zero ICC values do not indicate disagreement between raters but rather stem from the restricted range of scores due to a pronounced ceiling effect. This statistical artifact is well-recognized in studies where the evaluated material demonstrates uniformly high quality. Minor variations between ratings of 4 and 5 likely reflect subjective differences in professional emphasis rather than true divergence in evaluative standards. Thus, the descriptive agreement percentages, which exceeded 90% in three of four domains, may better represent the practical level of consensus among expert raters. This restricted score range is evident in Table 3, where nearly all ratings across all domains were either 5 or occasionally 4, leaving virtually no between-item variability for ICC estimation. Reporting medians and interquartile ranges confirms this pattern, underscoring that the apparent lack of ICC reliability reflects statistical artifact rather than evaluator disagreement. This phenomenon is not unique to our study; similar patterns have been reported in pedagogical research, psychological testing, and quality-of-care assessments when items cluster near the top of the rating scale. In such contexts, ICC values become mathematically unstable, making them a poor proxy for true agreement. Consequently, descriptive statistics and direct comparison of rating distributions provide a more accurate representation of evaluator alignment.

### 4.6. Clinical Utility and Future Directions

ChatGPT-3.5 shows promise as an adjunct in orthopaedic patient education, providing accurate and comprehensible responses that can support preoperative counseling and informed consent. However, it currently lacks the ability to personalize recommendations or consider individual patient factors such as comorbidities, which remain essential for comprehensive care. The evaluation of AI-generated patient information can be contextualized within established frameworks, such as the Common Framework for Patient-Reported Outcomes (PROs), which emphasizes the importance of tailoring information to patient needs and perspectives. Our findings indicate that ChatGPT-3.5 performs well in terms of relevance and clarity but may not fully meet the comprehensive standards required for individualized patient education. Recent research, including Moldovan et al. [28], highlights that AI tools are most effective when aligned with patient-centered frameworks, ensuring information is accessible and actionable for diverse populations. Our results support this, showing high ratings in clarity and relevance, but also revealing limitations in depth and individualization. Future studies should include broader evaluator groups, compare different AI models, and assess patient comprehension and satisfaction. Long-term research could clarify the impact of AI on clinical outcomes and patient adherence. Transparency in AI training and updates will be crucial for maintaining trust and accuracy in patient-facing applications. As AI becomes increasingly integrated into digital health pathways, establishing standardized guidelines for clinical use—including safety monitoring, hallucination auditing, and recommendations for appropriate clinical supervision—will be essential. Moreover, collaborative models that combine AI-generated information with clinician-led interpretation may offer the most effective balance between automation and individualized care. Ultimately, the role of LLMs in orthopaedics will depend not only on technological performance but also on thoughtful integration into clinical workflows, patient education strategies, and broader health literacy initiatives.

## 5. Conclusions

ChatGPT-3.5 demonstrated excellent performance in generating accurate, relevant, and comprehensible responses to patient questions about FAI and HAS. Despite ceiling effects limiting inter-rater reliability estimates, descriptive agreement and qualitative evaluation confirmed strong expert consensus and content quality. These findings support ChatGPT-3.5’s use as a supplementary patient education tool, provided appropriate clinical oversight is maintained to ensure safety, precision, and contextual accuracy in orthopedic patient communication.

## Figures and Tables

**Table 1:** Twenty most frequently asked patient questions about FAIS and HAS generated by ChatGPT-3.5.

No.	Question
1	What is FAIS?
2	What causes FAIS?
3	What are the symptoms of FAIS?
4	How is FAIS diagnosed?
5	Do I always need surgery for FAIS?
6	What types of FAIS are there?
7	What is hip arthroscopy and when is it used?
8	What happens during hip arthroscopy for FAIS?
9	What are the benefits of hip arthroscopy compared to open surgery?
10	What are the risks or complications of hip arthroscopy?
11	When can I return to sports or full activity?
12	Will my leg be shorter or longer after surgery?
13	Will I develop arthritis later if I have FAIS?
14	How do I prepare for hip arthroscopy?
15	What happens if I don’t have surgery?
16	How successful is hip arthroscopy for FAIS?
17	What factors affect the outcome of surgery?
18	Can I live a normal life after surgery?
19	How long will the benefits of surgery last?
20	What should I ask my surgeon before surgery?

**Table 2:** Mean ratings across evaluation domains.

Domain	Mean ± SD	ICC (2,1) [95% CI]	Exact Agreement (%)
Relevance	5.00 ± 0.00	not estimable	100
Accuracy	4.98 ± 0.11	0.00 [0.00–0.05]	90
Clarity	4.98 ± 0.11	0.01 [0.00-0.06]	90
Completeness	4.85 ± 0.24	0.03 [0.00–0.12]	70

**Table 3:** Complete evaluation ratings from both raters across all domains for each of the 20 questions.

Question	Relevance (Rater 1)	Relevance (Rater 2)	Accuracy (Rater 1)	Accuracy (Rater 2)	Clarity (Rater 1)	Clarity (Rater 2)	Completeness (Rater 1)	Completeness (Rater 2)
Question 1	5	5	5	5	5	5	5	5
Question 2	5	5	5	5	5	5	5	4
Question 3	5	5	5	5	5	5	5	5
Question 4	5	5	5	5	5	5	5	4
Question 5	5	5	4	5	4	5	5	5
Question 6	5	5	5	5	5	5	5	5
Question 7	5	5	5	5	5	5	5	5
Question 8	5	5	5	5	5	5	5	4
Question 9	5	5	5	5	5	5	5	4
Question 10	5	5	5	5	5	5	5	5
Question 11	5	5	5	5	5	5	5	4
Question 12	5	5	5	5	5	5	5	5
Question 13	5	5	5	5	5	5	5	5
Question 14	5	5	5	5	5	5	5	5
Question 15	5	5	5	5	5	5	5	4
Question 16	5	5	5	5	5	5	5	5
Question 17	5	5	5	5	5	5	5	5
Question 18	5	5	5	5	5	5	5	5
Question 19	5	5	5	5	5	5	5	5
Question 20	5	5	5	5	5	5	5	5

## Data Availability

All relevant data extracted and analyzed during this review are fully presented within the article, including in the corresponding tables and figures. ChatGPT-3.5-generated responses and complete evaluation ratings are available in the Supplementary Materials.

## Supplementary Materials

The following supporting information can be downloaded at: https://www.mdpi.com/article/10.3390/medicina61122080/s1, Table S1: Complete evaluation ratings from both raters across all domains for each of the 20 questions.

## Abbreviations

The following abbreviations are used in this manuscript:

AI	Artificial Intelligence
FAIS	Femoroacetabular Impingement Syndrome
HAS	Hip Arthroscopy
ICC	Intraclass Correlation Coefficient
LLMs	Large Language Models
SD	Standard Deviation
MDPI	Multidisciplinary Digital Publishing Institute
