# Supplementary material for: Assessing the Quality and Accuracy of ChatGPT-3.5 Responses to Patient Questions About Hip Arthroscopy
Source: Medicina (Kaunas). 2025 Nov 22;61(12):2080. doi: 10.3390/medicina61122080 (PMC12734417; doi:10.3390/medicina61122080)
Supplement: Supplementary file 1 [file medicina-61-02080-s001.zip › medicina-3993752-supplementary.pdf]

**Prompt provided to ChatGPT-5: “Create a list of the 20 most frequently asked questions by patients about femoroacetabular impingement syndrome and hip arthroscopy”**
**1. What is FAIS?**
FAIS stands for Femoroacetabular Impingement Syndrome. It’s a condition where the bones of the hip joint — the ball (femoral head) and the socket (acetabulum) — don’t fit together perfectly, causing them to rub abnormally during movement. Over time, this can damage the joint and lead to pain or limited motion.
**2. What causes FAIS?**
FAIS happens when the shape of the hip bones develops slightly differently than normal. Some people are born with this shape, while others develop it over time due to sports, repetitive hip movements, or past injuries. The abnormal contact causes irritation and damage to cartilage or the labrum (the ring of tissue around the socket).
**3. What are the symptoms of FAIS?**
Common symptoms include:<br>Groin or front-of-hip pain, especially during activity<br>Stiffness or limited range of motion<br>Clicking, locking, or catching sensations in the hip<br>Pain after sitting for long periods<br>Symptoms often start gradually and worsen over time.
**4. How is FAIS diagnosed?**
Diagnosis involves a physical exam, where your doctor checks your hip motion and pain points, and imaging tests, such as:<br>X-rays (to look at bone shape)<br>MRI or MR arthrogram (to check soft tissues like cartilage and labrum)<br>CT scans (for detailed bone imaging if needed)
**5. Do I always need surgery for FAIS?**
No. Many people improve with non-surgical treatments like:<br>Physical therapy to improve hip strength and motion<br>Activity modification (avoiding movements that trigger pain)<br>Anti-inflammatory medications or occasional injections<br>Surgery is considered when symptoms persist despite these measures.
**6. What types of FAIS are there?**
There are three main types:

Cam type – a bump on the femoral head (ball) that causes rubbing.<br>Pincer type – extra bone on the acetabulum (socket) that leads to pinching.<br>Mixed type – both cam and pincer features.<br>Knowing the type helps guide treatment and surgical planning.
**7. What is hip arthroscopy and when is it used?**
Hip arthroscopy is a minimally invasive surgery where small instruments and a camera are inserted through tiny incisions to repair damage inside the hip joint. It's used when conservative treatments fail and imaging confirms FAIS or related labral tears.
**8. What happens during hip arthroscopy for FAIS?**
During the procedure:<br>You're given anesthesia.<br>The surgeon makes small incisions and inserts a camera and instruments.<br>Extra bone is shaved down (to correct impingement).<br>The labrum and cartilage are repaired or smoothed.<br>The incisions are closed with small stitches.<br>Surgery usually takes 1–2 hours.
**9. What are the benefits of hip arthroscopy compared to open surgery?**
Smaller incisions and less tissue damage<br>Faster recovery and less pain afterward<br>Lower risk of complications<br>Quicker return to daily activities and sports<br>Better cosmetic results (smaller scars)
**10. What are the risks or complications of hip arthroscopy?**
Though uncommon, possible risks include:<br>Temporary numbness or tingling around the thigh<br>Infection or bleeding<br>Blood clots (rare)<br>Persistent pain or stiffness<br>Incomplete relief if arthritis or advanced damage is present
**11. When can I return to sports or full activity?**
Most patients:<br>Walk with crutches for 1–3 weeks<br>Return to light activity in 6–8 weeks

Resume sports in about 4–6 months<br>Full recovery can vary depending on the type of repair and individual healing.
**12. Will my leg be shorter or longer after surgery?**
No. Hip arthroscopy doesn’t change leg length. Some people feel a temporary difference due to muscle tightness or swelling, but this resolves with recovery and physical therapy.
**13. Will I develop arthritis later if I have FAIS?**
FAIS increases the risk of arthritis if untreated because of ongoing joint damage. Early diagnosis and treatment—including surgery when needed—can help protect the hip and possibly delay or prevent arthritis.
**14. How do I prepare for hip arthroscopy?**
Preparation may include:<br>Pre-surgery physical therapy to strengthen muscles<br>Stopping certain medications (like blood thinners)<br>Arranging help at home for the first few days<br>Fasting the night before surgery<br>Your surgeon’s team will provide detailed instructions.
**15. What happens if I don’t have surgery?**
Without surgery, symptoms may persist or worsen. Continued impingement can lead to more cartilage and labral damage, and eventually to early hip arthritis. However, if symptoms are mild, careful management and lifestyle changes can sometimes control them.
**16. How successful is hip arthroscopy for FAIS?**
Studies show 80–90% of patients have significant pain relief and improved function after surgery. Success depends on factors like age, amount of joint damage, and adherence to rehabilitation..
**17. What factors affect the outcome of surgery?**
Outcomes are best when:<br>There is minimal arthritis before surgery<br>The labrum can be repaired rather than removed<br>Patients are under 40–45 years old<br>Rehabilitation is followed closely<br>Smoking, obesity, and advanced cartilage damage can reduce success.
**18. Can I live a normal life after surgery?**
Yes. Most people return to regular daily activities, work, and sports. With proper recovery and lifestyle choices, many patients live pain-free and maintain normal hip function for years.

19. How long will the benefits of surgery last?
For most, improvements last many years—often 10 or more—especially if arthritis is not already advanced. Maintaining hip strength and avoiding repetitive high-impact activities can help preserve results.
**20. What should I ask my surgeon before surgery?**
Consider asking:<br>What type of impingement do I have?<br>What are my treatment options?<br>What is your experience with hip arthroscopy?<br>What is my expected recovery time?<br>What are the risks in my specific case?<br>How can I prepare for the best outcome?
